# Supplementary material for: Massively Parallel Imitation Learning of Mouse Forelimb Musculoskeletal Reaching Dynamics
Source: ArXiv. 2025 Nov 26:arXiv:2511.21848v1. Preprint. [Version 1] (PMC12676374)
Supplement: 1 [file NIHPP2511.21848V1-supplement-1.pdf]

6 Supplemental Materials

Table 1: Data types and formats for rollouts

Name	Description	Format	Size
Multicamera Video	frames × 3	.mp4	∼1.5 GB
2D Pose Estimation	frames × nodes × 2	.h5, .slp	∼12 MB
Multi-Camera Calibration	Intrinsics, extrinsics, distortions	.toml	∼1 KB
3D Pose Estimation	frames × nodes × 3	.h5	∼8 MB
Inverse Kinematics	Joint angles over time from STAC-MJX	.h5	∼2 MB
Registration Offsets	Offsets between pose estimation and MuJoCo model	.h5	∼10 MB
EMG Observations	Biceps and triceps recorded in the lab over time	.csv	∼128 MB
Trial info	Indexes for trial start and reach start.	.csv	∼1 KB
**Rollouts (single file; multiple keys)**		.h5	∼32 MB
Latent Activations	4 latent dimensions over time		
Encoder Activations	3 layers of 512 activations over time		
Decoder Activations	3 layers of 512 activations over time		
Simulated Muscle Activations	9 muscle actuators over time		
Simulated Kinematics	Four joints over time		

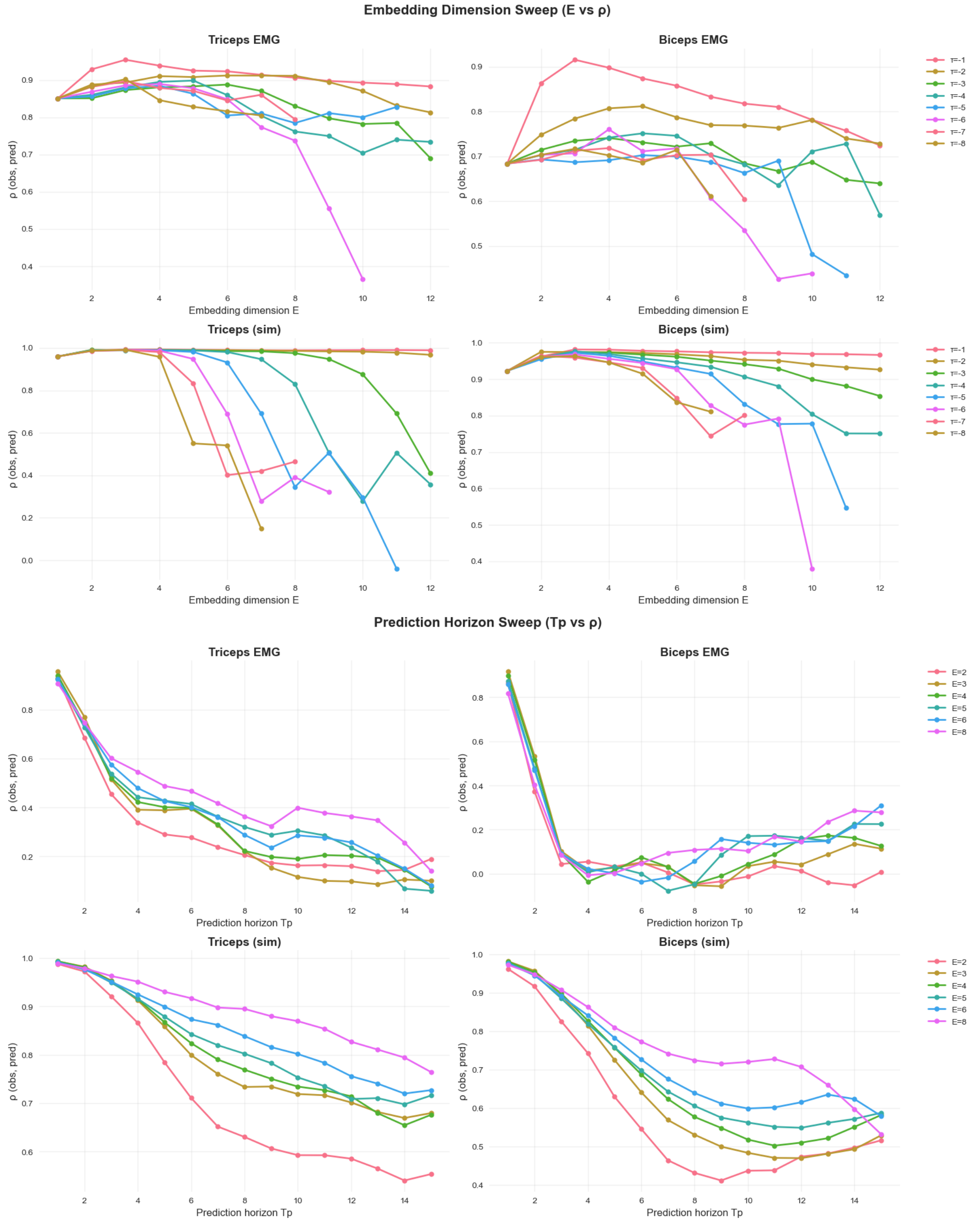

Figure 4: PyEDM parameter search. Embedding dimension and tau sweep with performance measured by simplex rho for the simulated muscle activity and EMG signals find an optimal tau of -1 and optimal embedding dimension of 3. Prediction horizon search for the simulated muscle activity and EMG signals show the presence of a nonlinearity.

PPO Params	Joint Only	Physics Aware
num envs	4096	4096
batch size	1024	1024
num minibatches	8	8
learning rate	1.00E-04	1.00E-04
clipping epsilon	0.2	0.2
discounting	0.95	0.95
entropy cost	0.001	0.001
unroll length	20	20
kl weight	0.00001	0.00001
**Network Params**		
encoder layer sizes	[512,512,512]	[512,512,512]
decoder layer sizes	[512,512,512]	[512,512,512]
critic layer sizes	[512,512,512]	[512,512,512]
intention size	4	4
**Sim Params**		
sim dt	0.00125	0.00125
ctrl dt (sim_dt * steps...)	0.0025	0.0025
solver	CG	CG
iterations	6	6
ls iterations	6	6
**Reward Params**		
pos exp scale	0.0	0.0
quat exp scale	0.0	0.0
joint exp scale	0.2	0.2
end eff exp scale	0.0	0.0
body pos exp scale	0.0	0.0
joint vel exp scale	0.0	0.0
pos weight	0.0	0.0
quat weight	0.0	0.0
joint weight	5	5
end eff weight	0.0	0.0
body pos weight	0.0	0.0
joint vel weight	0.0	0.0
control cost	0.0	0.15
control difference cost	0.0	0.0
energy cost	0.0	0.01
variance cost	0.0	0.0
variance window	0.0	0.0

Table 2: Training configuration parameters for Joint-Only and Physics-Aware mouse arm models.
